# Mechanisms of Low-Temperature Nitridation Technology on a TaN Thin Film Resistor for Temperature Sensor Applications

**DOI:** 10.1186/s11671-016-1480-z

**Published:** 2016-06-01

**Authors:** Huey-Ru Chen, Ying-Chung Chen, Ting-Chang Chang, Kuan-Chang Chang, Tsung-Ming Tsai, Tian-Jian Chu, Chih-Cheng Shih, Nai-Chuan Chuang, Kao-Yuan Wang

**Affiliations:** Department of Electrical Engineering, National Sun Yat-Sen University, Kaohsiung, Taiwan; Department of Physics, National Sun Yat-Sen University, Kaohsiung, Taiwan; Department of Materials and Optoelectronic Science, National Sun Yat-Sen University, Kaohsiung, Taiwan; R&D Department, Walsin Technology Co, Kaohsiung, Taiwan

**Keywords:** TaN, Thin film resistor, Temperature coefficient of resistance, SCCO_2_

## Abstract

In this letter, we propose a novel low-temperature nitridation technology on a tantalum nitride (TaN) thin film resistor (TFR) through supercritical carbon dioxide (SCCO_2_) treatment for temperature sensor applications. We also found that the sensitivity of temperature of the TaN TFR was improved about 10.2 %, which can be demonstrated from measurement of temperature coefficient of resistance (TCR). In order to understand the mechanism of SCCO_2_ nitridation on the TaN TFR, the carrier conduction mechanism of the device was analyzed through current fitting. The current conduction mechanism of the TaN TFR changes from hopping to a Schottky emission after the low-temperature SCCO_2_ nitridation treatment. A model of vacancy passivation in TaN grains with nitrogen and by SCCO_2_ nitridation treatment is eventually proposed to increase the isolation ability in TaN TFR, which causes the transfer of current conduction mechanisms.

## Background

With the rapid development of Internet of Things (IOT) technology, the improvement of sensor technologies, such as temperature sensors, gas sensors, and optical sensors, is required to integrate with memory devices [1–23], logic devices, and passive devices [24–28] in one chip in the future. In addition to the volume of traditional sensor devices being large, the materials used in the manufacture need to be processed at a high temperature, which cannot be compatible with the back end of the line process of integrated circuit (IC) manufacturing technology. Therefore, low-temperature and IC technology-compatible materials should be developed for sensor devices and IOT technology. Tantalum nitride is a mechanically hard, chemically inner, and corrosion-resistant material and has good shock/heat-resistant properties. These properties make the material attractive for many industrial applications.

A supercritical phase is peculiar with its characteristics of high penetration of gas and solubility of liquid [29–39]. The supercritical ammonia fluid has nitridation ability for materials. In order to achieve supercritical ammonia at lower temperature, little ammonia was added into supercritical CO_2_ fluids, from which the liquid ammonia can attain to the supercritical fluid phase due to the phase close to an ideal solution.

In this study, a tantalum nitride (TaN) thin film resistor was fabricated to investigate improvement of temperature sensitivity with supercritical carbon dioxide (SCCO_2_) nitridation technology through current-voltage measurement and analysis. The current fitting methods were applied so as to analyze the physical mechanisms of carrier conduction in TaN films with SCCO_2_ nitridation treatment. Conduction current fitting together with vary-temperature current-voltage measurement data were thoroughly investigated, from which current conduction mechanisms were determined. Finally, a molecular reaction model was proposed to explain the influence of the SCCO_2_ nitridation process on the current conduction mechanisms in the TaN thin film resistor. We believe that the temperature sensitivity of the TaN thin film can be improved by SCCO_2_ nitridation technology at lower temperature.

### Methods

**Fig. 1:**
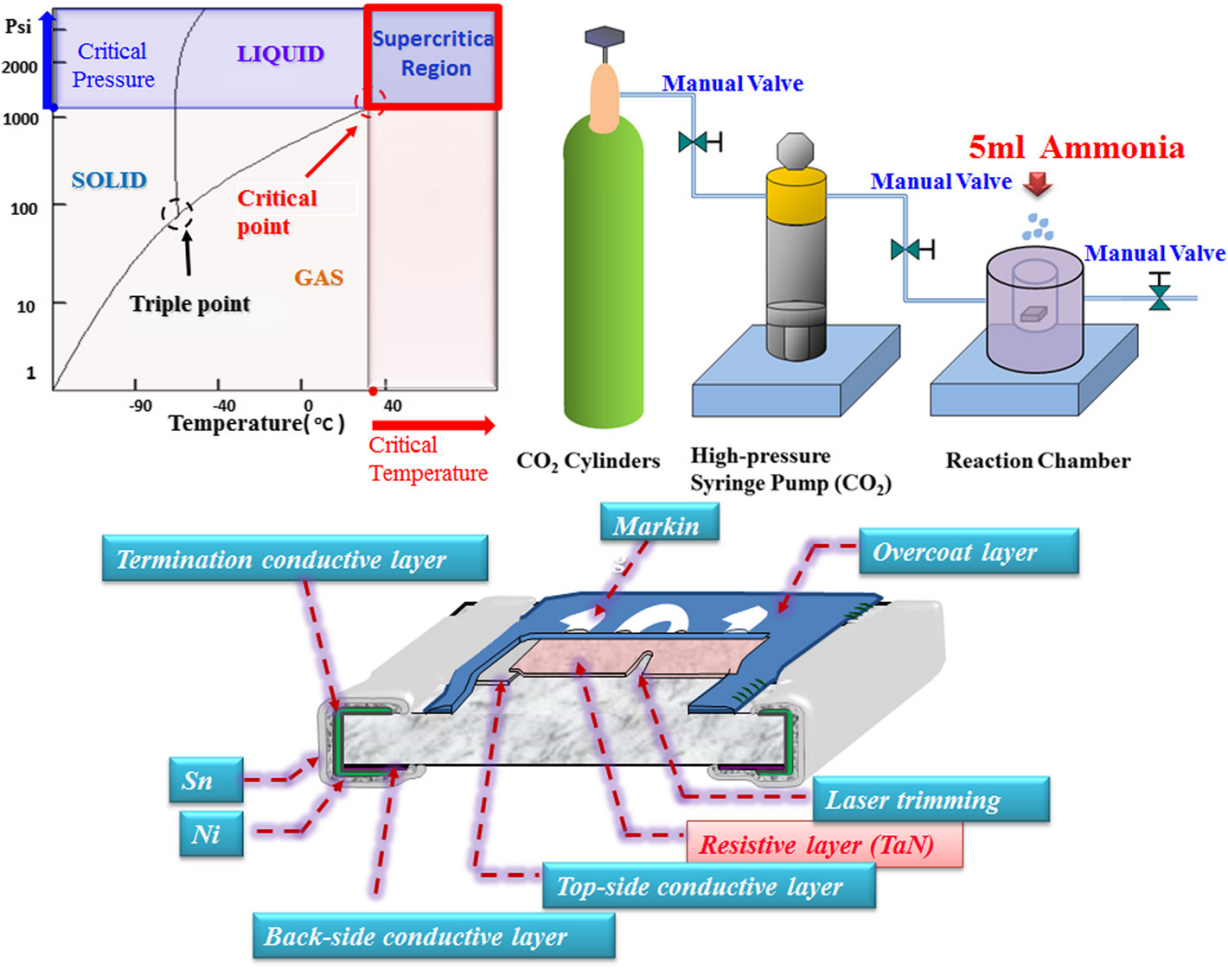
The schematic diagram of supercritical CO_2_ fluid systems and the TaN thin film resistor chip schematic structure

The experimental thin film temperature sensing resistor devices (the bottom scheme of Fig. 1) were prepared as follows: Firstly, the conductor silver material was printed on an alumina substrate. Then 150-nm TaN films were deposited on the silver-printed substrate by DC sputtering with a Ta target in the Ar/N_2_ mixed gas ambient. After that, the TaN films were put into a reactive chamber of supercritical fluid system with a 165-ml chamber size (Ying-Kwan Bio Tech Co., Ltd., Taipei, Taiwan). Then the SCCO_2_ fluid mixed with 5 ml ammonia solution which is adsorbed on zeolite was syringed into the reactive chamber to treat the samples as shown in the top scheme of Fig. 1. Therefore, the ammonia will be solved into SCCO_2_ fluids with a mole concentration of 1.7 M in the reactive chamber. During the treatment, the ammonia-mixed supercritical CO_2_ fluids were heated and pressured to 120 °C and 3000 psi, respectively, in the stainless steel chamber of supercritical fluid system for 1 h. In order to conduct the electrical measurement and analysis of the TaN thin film resistor for temperature sensor application, a snake-type pattern was realized by the laser-trimming process using green laser to control resistance value. The entire electrical measurements of devices were performed using the Agilent B1500 semiconductor parameter analyzer.

## Results and Discussion

**Fig. 2:**
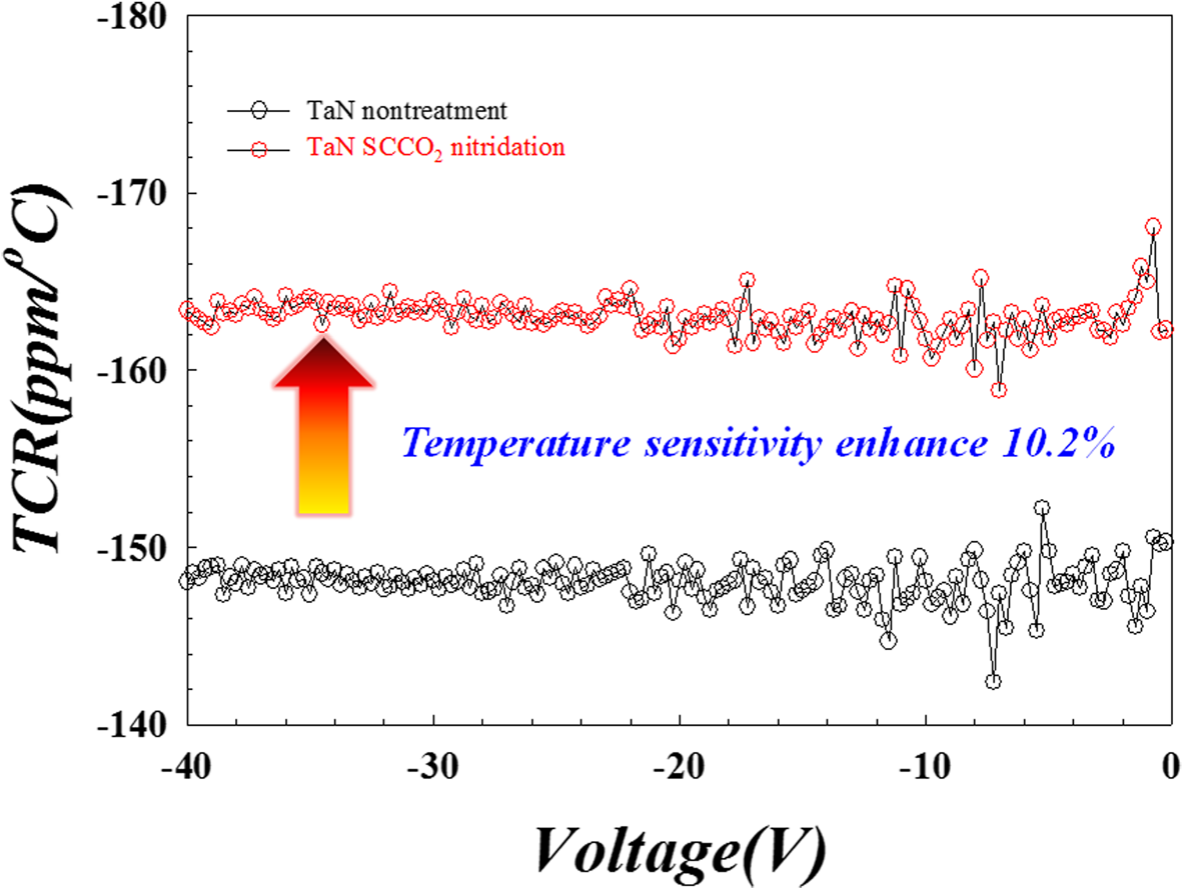
TCR value of the TaN thin film resistor. The TCR value was calculated from the relationship of resistance of the TaN thin film resistor at different temperatures (DC I-V sweeping voltage was set from 0 to −40 V). The temperature sensitivity of the TaN thin film resistor was enhanced about 10.2 % through SCCO_2_ nitridation technology

The DC current-voltage (I-V) sweeping was applied to investigate the electrical characteristics of the TaN thin film resistor before and after SCCO_2_ nitridation treatment. To testify the temperature sensitivity of the TaN thin film resistor, vary-temperature I-V measurement was conducted at variable temperature from 30 to 80 °C. The temperature coefficient of resistance (TCR) value is defined as the ratio of resistance change between different temperatures, TCR = (*R*_1_ − *R*_0_)/*R*_0_*(1/*T*_1_ − *T*_0_) × 10^6^ (ppm/°C), where *T*_0_ is 30 °C, *T*_1_ is 80 °C, *R*_0_ is the resistance value at 30 °C, and *R*_1_ is the resistance value at 80 °C. After converting the I-V curve to TCR values, we found that the temperature sensitivity of the TaN thin film was enhanced about 10.2 % through SCCO_2_ nitridation technology, as shown in Fig. 2. If the TCR value is negative, the resistance value of the TaN thin film has negative correlation with temperature. The higher absolute value of TCR represents greater change amount with temperature.

**Fig. 3:**
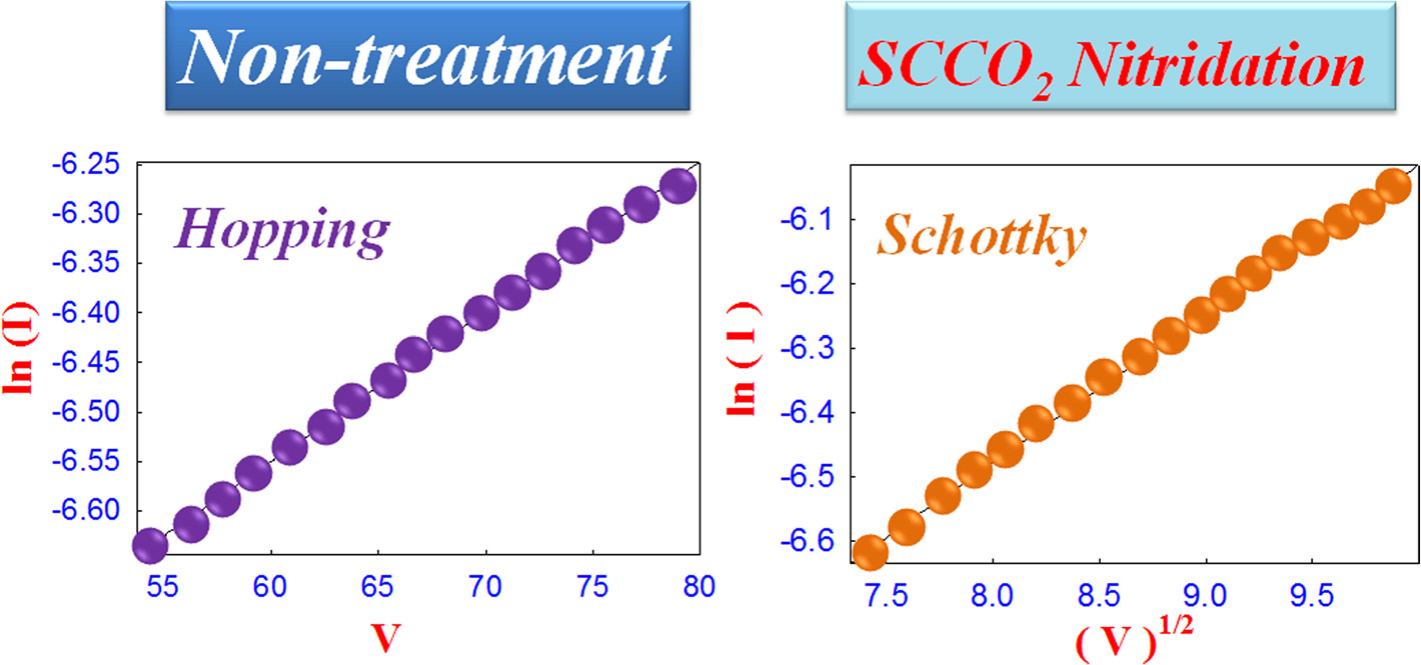
Current conduction mechanism fitting of TaN thin film resistors with and without SCCO_2_ nitridation treatment

To investigate the influence of SCCO_2_ nitridation on electrical properties of the TaN thin film, we analyzed the current conduction mechanism of the TaN thin film resistor (TFR) device with and without SCCO_2_ nitridation treatment as shown in Fig. 3. The relationship in curve of ln(*I*) versus the applied voltage (*V*) for the treated TaN TFR device is linear. According to the equation of hopping conduction, *J* = *qNaυ*_0_*e*^−*qφT*/*kT*^*e*^*qaV*/2*dkT*^, where *N*, *a*, *φ*, *T* , *υ*_0_, and *d* are density of space charge, mean of hopping distance, barrier height of hopping, intrinsic vibration frequency, and film thickness, respectively. Therefore, the current conduction mechanism of the TaN TFR without SCCO_2_ nitridation treatment is dominated by hopping conduction mechanism. After SCCO_2_ nitridation treatment on the TaN TFR device, the relationship in the curve of ln(*I*/*T*^2^) versus the square root of the applied voltage (*V*^1/2^) is linear. According to the formula of the Schottky emission, $$ J={A}^{**}{T}^2 \exp \left[\frac{\hbox{-} q\left({\phi}_B\hbox{-} \sqrt{\raisebox{1ex}{$qV$}\!\left/ \!\raisebox{-1ex}{$4\pi {\varepsilon}_id$}\right.}\right)}{kT}\right] $$, where A** is the Richardson constant, *d* is the film thickness, and (ϕ_B_) is activation energy barrier height, the carrier conduction mechanism of the TaN TFR was transferred to a Schottky emission after SCCO_2_ nitridation treatment.

**Fig. 4:**
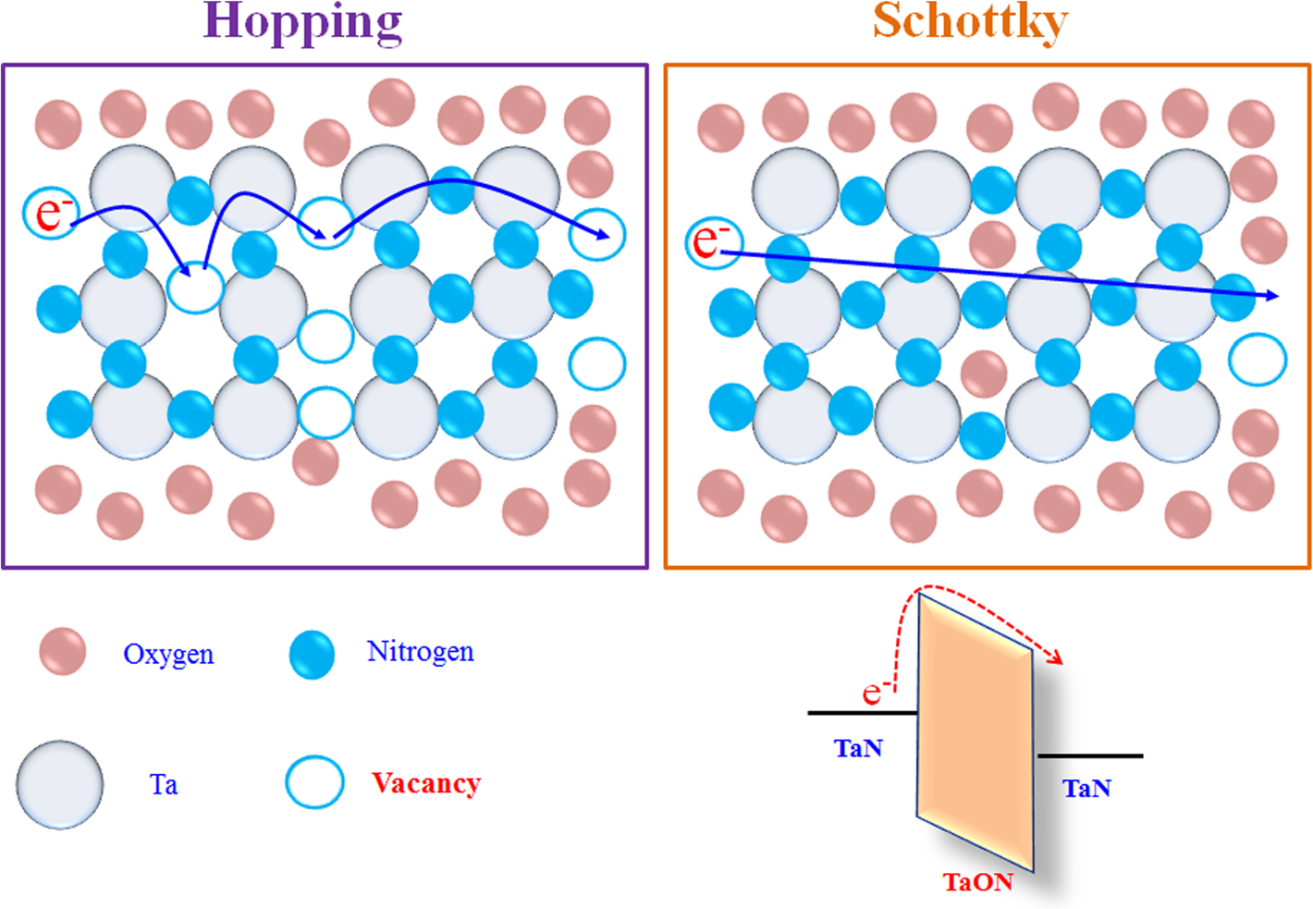
The schematic diagram of nitrogen passivation in TaN thin film resistor with SCCO_2_ nitridation technology

Based on the results of electrical analyses, a carrier conduction model of the TaN TFR with SCCO_2_ nitridation treatment was proposed in Fig. 4. As the TaN thin film was deposited at low temperature by DC-sputtering technology, there are many vacancies existing in the TaN thin film. When the voltage is applied on the as-deposited TaN TFR, the carrier will be transported by hopping through the vacancies, resulting in the current conduction mechanism of the as-deposited TaN TFR being dominated by hopping conduction. Because the temperature sensitivity is low for hopping conduction mechanism, the absolute TCR value of the as-deposited TaN TFR is small. After the as-deposited TaN TFR was treated by SCCO_2_ nitridation technology, the nitrogen atoms will penetrate into the TaN thin film to passivate the vacancies in grain boundary of the TaN grains, resulting in an insulating tantalum oxynitride (TaON) layer formed between the TaN grains. The TaON layer will increase the thermal activation energy barrier height of carrier transport, leading to the current conduction mechanism of the SCCO_2_ nitridation-treated TaN TFR dominated by the Schottky emission. Because the Schottky conduction is due to emission of electron cross-activation energy barrier height, the current conduction of the SCCO_2_ nitridation-treated TaN TFR is sensitive to temperature, resulting in the improvement of temperature sensitivity of the TaN TFR.

## Conclusions

In conclusion, the vacancies between the TaN grains were successfully passivated by SCCO_2_ nitridation technology to form an insulating TaON layer. After SCCO_2_ nitridation treatment, the carrier conduction mechanism of the TaN TFR transforms from hopping conduction to a Schottky emission conduction due to the formation of the TaON layer between TaN grain boundary, which causes the enhancement of temperature sensitivity of the TaN TFR. It is believed that the low-temperature SCCO_2_ nitridation treatment is a promising technology for high-temperature sensitivity sensor applications.
